# Serum Cytokine Profiling Identifies Axl as a New Biomarker Candidate for Active Eosinophilic Granulomatosis With Polyangiitis

**DOI:** 10.3389/fmolb.2021.653461

**Published:** 2021-04-27

**Authors:** Jianjuan Ma, Cong Dong, Shushan Wei, Minzhi Qiu, Penghui Wu, Changxing Ou, Bomeng Zhang, Xueyan Zhang, Jie Yan, Qingling Zhang, Nanshan Zhong

**Affiliations:** ^1^Department of Pathophysiology, School of Basic Medical Sciences, Guizhou Medical University, Guiyang, China; ^2^Pulmonary and Critical Care Medicine, Guangzhou Institute of Respiratory Health, National Clinical Research Center for Respiratory Disease, National Center for Respiratory Medicine, State Key Laboratory of Respiratory Diseases, The First Affiliated Hospital of Guangzhou Medical University, Guangzhou, China; ^3^Department of Pediatric Hematology, Affiliated Hospital of Guizhou Medical University, Guiyang, China; ^4^School of Basic Medical Sciences, The Second Affiliated Hospital, State Key Laboratory of Respiratory Disease, Guangdong Provincial Key Laboratory of Allergy & Clinical Immunology, Guangzhou Medical University, Guangzhou, China; ^5^The Second Affiliated Hospital, State Key Laboratory of Respiratory Disease, Guangdong Provincial Key Laboratory of Allergy & Clinical Immunology, Guangzhou Medical University, Guangzhou, China

**Keywords:** eosinophilic granulomatosis with polyangiitis, antibody array, biomarkers, active, serum

## Abstract

**Background:** Eosinophilic granulomatosis with polyangiitis (EGPA) prognosis is generally favorable and is treated with combined corticosteroids/immunosuppressor(s) therapy. However, disease flares increase the number of clinical visits. Therefore, discovering new serum biomarkers for early identification of active EGPA is crucial.

**Objective:** To identify reliable serum biomarkers to measure EGPA activity.

**Methods:** The expression of 160 proteins was compared in sera from 15 inactive and 13 active EGPA patients by antibody-based microarray. Network-based analysis identified patterns in the different groups. Differentially expressed proteins (DEPs) in active disease were identified, and the correlation between their serum levels and clinical parameters was assessed. DEPs were further analyzed for GO enrichment and KEGG pathways. Finally, DEP marker candidates were validated by ELISA and Bio-plex as well as against a second cohort of 22 inactive and 18 active EGPA patients.

**Results:** The active group presented higher peripheral and sputum eosinophil counts, FeNO, and FEV1 (% predicted) (*P* < 0.05). Network-based analysis showed scattered expression patterns in active subjects, but no significant bias in inactive subjects. Significant differences were observed in serum levels of 19 candidate markers, all of which were higher in active EGPA (*P* < 0.05). KEGG analysis indicated that DEPs were mainly involved in the MAPK, PI3K-Akt, RAS and Rap1 related pathways. Nine out of 19 candidate markers were positively correlated with peripheral eosinophil counts including FGF-7, SCF, GDNF, β-NGF, IGFBP-4, Axl, PIGF, Insulin, NT-4, ErbB3, OPN and BMP-4 (*r* = 0.693, *r* = 0.692, *r* = 0.687, *r* = 0.683, *r* = 0.671, *r* = 0.606, *r* = 0.571, *r* = 0.570, *r* = 0.516, respectively; *P* < 0.05), while two, CD14 and MCP-3, were negatively correlated (*r* = −0.644 and *r* = −0.515; *P* < 0.05). The higher expression of Axl, OPN, HCC-4, GDNF, and MCP-3 in active EGPA subjects was confirmed by ELISA and Custom Multiplex Bio-plex analyses.

**Conclusion:** The serum protein profiles were significantly different between active and inactive EGPA. The expression of the candidate proteins correlated with peripheral blood eosinophil count. Serum Axl, OPN, HCC-4, GDNF, and MCP-3 levels were consistently higher in active EGPA, independent of the assessment methods. Finally, Axl had the largest AUC, indicating that this cytokine may serve as novel biomarker for the diagnosis of active EGPA.

## Introduction

Eosinophilic granulomatosis with polyangiitis (EGPA), formerly called Churg-Strauss syndrome, is a systemic necrotizing vasculitis affecting small- and medium-size vessels; it is characterized by asthma, peripheral eosinophilia, eosinophil-rich inflammation, and vascular and/or extravascular granulomas. Since 1951, when EGPA was first reported by Churg and Strauss, there have been several definitions and classification criteria for EGPA (Churg and Strauss, 1951). In 1990, the American College of Rheumatology (ACR) proposed the first EGPA classification criteria including the following six items (Lightfoot et al., 1990; Masi et al., 1990): (1) asthma; (2) paranasal sinus abnormality; (3) peripheral blood eosinophilia (>10%); (4) unfixed pulmonary infiltration; (5) mononeuropathy or polyneuropathy; and (6) extravascular eosinophils on histology. When four or more items are satisfied, a patient can be classified as EGPA. The sensitivity and specificity of the 1990 ACR criteria for EGPA have been reported were 85% and 99.75% respectively (Masi et al., 1990).

However, there are no definitive diagnosis criteria for EGPA. EGPA diagnosis remains clinical and is based on the existence of vasculitis. Moreover, discriminating disease activity from worsening underlying asthma is challenging. Thus, biomarkers to diagnose EGPA disease flares would be extremely valuable. Further, to improve EGPA management, researchers have attempted to identify biomarkers that discriminate active and inactive EGPA. Some studies have established associations between common laboratory readouts, e.g., serum IgE, erythrocyte sedimentation rate and C-reactive protein (CRP) (Peter C. Grayson et al., 2015), and disease activity, but these have limitations as longitudinal biomarkers to monitor disease activity. In addition, the eosinophil cationic protein was correlated with disease activity in small series. Other studies examined several novel biomarkers, e.g., CCL17/TARC (Dallos et al., 2010; Dejaco et al., 2015), IgG4 (Augusto Vaglio et al., 2012; Dejaco et al., 2015) and CCL26/eotaxin-3 (Polzer et al., 2008; Dejaco et al., 2015; Zagvozdkina et al., 2016), but their use for routine diagnosis has not yet been implemented. These findings suggest that novel biomarkers to monitor disease activity in EGPA are still needed.

High throughput protein expression studies, such as microarray analyses, are increasingly used to identify changes in protein profiles during the onset and progression of complex diseases. In this study, we integrated microarray data from EGPA and sought to identify protein expression profiles and signaling pathways that can mark pathogenetic changes in active EGPA. This study led to the identification of new diagnostic markers and therapeutic targets.

## Methods

### Study Design and Subjects

In the current study, we conducted a prospective observational study in which 2 groups of patients were recruited, including 31 patients with active EGPA, and 37 patients with inactive EGPA, all treated at the Department of Allergy and Clinical Immunology at The First Affiliated Hospital of Guangzhou Medical University, China. Among them, an exploration subgroup included 15 inactive patients and 13 active patients. Other 22 inactive patients and 18 active patients were recruited in the validation study.

All EGPA subjects met four or more ACR items (Masi et al., 1990), including (1) asthma; defined as a history of wheezing or diffuse high- pitched rales on expiration; (2) peripheral blood eosinophilia (>10%); (3) mononeuropathy or polyneuropathy; defined as the development of mononeuropathy, multiple mononeuropathies, or polyneuropathy (i.e., glovel stocking distribution) attributable to a systemic vasculitis; (4) unfixed pulmonary infiltration; defined as migratory or transitory pulmonary infiltrates on radiographs (not including fixed infiltrates), attributable to a systemic vasculitis; (5) paranasal sinus abnormality; defined as a history of acute or chronic paranasal sinus pain or tenderness or radiographic opacification of the paranasal sinuses and (6) extravascular eosinophils on histology; defined as biopsy including artery, arteriole, or venule, showing accumulations of eosinophils in extravascular areas. All EGPA subjects had vasculitis manifestations and met ACR criteria. Disease activity was assessed at diagnosis using the original 1994 Birmingham Vasculitis Activity Score (BVAS) (Luqmani et al., 1994) and was recorded in the database. Patients with a score under 15 were recruited to the inactive group, whereas they joined the active group above a score of 15. All subjects underwent a standardized clinical assessment, including pulmonary function test, sputum induction, inflammatory cell differentiation test, allergy status or serum(Se)-specific-IgE measurement, fractional exhaled nitric oxide (FeNO), anti-neutrophil cytoplasmic antibodies (ANCA) test and complete blood count (CBC).

The study was approved by the Ethics Review Board of the First Affiliated Hospital of Guangzhou Medical University [Medical research ethics review 2018,No.35]. All participants provided written informed consent.

### Lung Function

Lung function was measured by spirometer (MasterScreen PFT; Jaeper^TM^, CareFusion, Hoechberg, Germany), in accordance with the American Thoracic Society/European Respiratory Society guidelines (Miller et al., 2005). Parameters, including percent of predicted forced vital capacity (FVC%) and forced expiratory volume in 1 second of predicted (FEV_1_%), percent of predicted peak expiratory flow (PEF%) and maximum mid expiratory flow (MMEF75/25), were recorded.

### Blood Sample

Five milliliters of venous blood sample were collected into vacutainer tubes containing no additive. Sera were prepared by centrifugation and stored at −80°C for protein microarray analysis, ELISA and Bio-Plex assay.

### Protein Microarray Analysis

RayBiotech Human Cytokine Antibody Array 3000 (RayBiotech, Inc., Cat# QAH-CAA-3000) was used to analyze serum protein profiles. This antibody array simultaneously detects 160 human cytokines shared between 4 non-overlapping arrays (Supplementary Table 1). The samples were read by the Guangzhou Biotech Company. The experimental procedure was carried out in accordance with the manufacturer’s instructions. Briefly, antibody pre-coated array membranes were incubated with coating buffer for 30 min. The blocking buffer was then decanted and replaced with dilutions of the samples, for overnight incubation at 4°C under shaking. The next day, the membranes were washed and incubated with a mix of biotin-conjugated antibodies for 2 h. The biotin-conjugated antibodies were removed, and Streptavidin-Fluor was added to each subarray. The incubation chambers were covered with adhesive film and incubated for 2 h. After washing, signals were read with a GenePix 4000B system (Axon Instruments, Foster City, CA, United States). GenePix Pro 6.0 software (Axon Instruments) was used for densitometric analysis of the spots. The values were normalized to the signals obtained for the positive controls of each sample. The total normalized fluorescence values of the replicates were averaged and are expressed as the fold increase compared to the control samples.

### Gene Ontology and KEGG Pathway Analysis

The Database for Annotation, Visualization and Integrated Discovery (DAVID^1^) is an online biological information database for gene functional analysis (Huang et al., 2007). To predict the possible functions of the overlapping differentially expressed proteins (DEPs) and their neighboring genes, and the potential pathways in which they intervene, we performed Gene Ontology (GO) enrichment and KEGG pathway analyses in DAVID. *P* < 0.05 was considered statistically significant.

### ELISA

ELISA kits (Thermo/Invitrogen Carlsbad, CA, United States) were used to validate the results obtained from the antibody arrays, following the manufacturer’s instructions. Briefly, serum samples were diluted at different dilution factors based on individual serum biomarker characteristics. Samples were coated on the plates for 1 to 2.5 h at room temperature. The plates were washed, and a biotin-conjugated antibody specific for each kit was added and incubated for 1 h under gentle shaking. Horse Radish Peroxidase (HRP)-conjugated streptavidin was added to catalyze the 3,3′,5,5′-Tetramethylbenzidine (TMB) chromogenic reagent. Finally, the catalytic reaction was stopped by addition of sulfuric acid. At each step, the reactional volume in each well was of 100 μl. Finally, the OD450 was read using a microplate reader (Multiskan GO, Thermo Scientific, Carlsbad, CA, United States).

### Bio-Plex Assay

The levels of 13 cytokines were assessed in the sera by the Custom Multiplex Kit featuring Axl, GDNF, HB-EGF, MCP-3, NGF beta, PDGF-BB, PlGF-1 and SCF (Thermo/invitrogen Carlsbad, CA, United States; Cat#PPX-08), and the Custom Multiplex Kit featuring BMP-4, ErbB3, IGFBP-4, Insulin and NT-4 (Thermo/invitrogen Carlsbad, CA, United States; Cat# LSAHM-05). The experimental procedure was carried out according to the manufacturer’s instructions. Briefly, beads coated with capture antibodies were incubated with premixed standards or sample supernatants for 120 min at room temperature under 500-rpm agitation. Following incubation, premixed detection antibodies were added and incubated for 30 min at room temperature under 500-rpm agitation. After washing, Phycoerythrin (PE)-conjugated streptavidin was added and incubated as before. After washing, the beads were resuspended in Bio-Plex cytokine assay buffer and read on the Bio-Plex 200 system using Low PMT setting. Data were analyzed with Bio-Plex Manager^TM^ software version 2.0.

### Statistical Analysis and Visualization

Statistical analysis was performed using the SPSS software package (version 22.0; IBM Corp., Armonk, NY, United States). Continuous variables are expressed as number (%) or means ± standard deviation (SD). Comparisons between groups were made using the *t*-test or Mann-Whitney *U*-test for continuous endpoints, and the χ^2^ test for categorical endpoints. *P*-values were corrected using the Benjamini-Hochberg false discovery rate (FDR). Correlations were assessed using Pearson’s or Spearman’s correlation test. A *P*-value of < 0.05 was considered significant.

Network analysis based on the Spearman analysis was performed using Gephi V0.9.2 open source software^2^ output showing the 160 serum soluble proteins from the different subjects. Each serum soluble protein is represented by a specific node whose color represents a specific cluster. The size of the node is proportional to the sum of the edges that connects to them. Edges connecting nodes represent statistically significant correlations (*P* < 0.05). The edge’s thickness represents the strengths of their association (Spearman’s correlation). We used the existence of modules represented by highly interlinked to pological clusters in the network using the computational algorithm proposed by Blonde et al. (Lancichinetti and Fortunato, 2009) and included in the Gephi statistical module. Modularity is the fraction of the edges that fall within the given groups of nodes minus the expected fraction if the edges were distributed at random. The value of the modularity lies in the range of −1 to 1. If positive then the number of edges within groups exceeds the number expected on the basis of chance.

## Results

### Patient Characteristics

After screening, we recruited 37 patients with inactive EGPA and 31 patients with active EGPA. All subjects had a definitive EGPA diagnosis, presented more than two organs involvement, and tested negative for ANCAs. There were no significant clinical differences among all EGPA subjects regarding lung function indices (FVC (% predicted), FEV1/FVC, PEF (% predicted), MMEF75/25) and organ involvement (*P* > 0.05). However, the EGPA active group presented higher peripheral and sputum eosinophil counts, FeNO and FEV1 (% predicted) (*P* = 0.000, *P* = 0.000, *P* = 0.015, *P* = 0.030, respectively). The demographics and characteristics of the subjects are shown in Table 1.

**TABLE 1:** Clinical manifestations in patients with EGPA.

**Subjects’ characteristics**	**Inactive (*n* = 31)**	**Active (*n* = 37)**	***P*-value**
Age, mean ± SD years*	42.1 ± 12.7	42.8 ± 18.1	0.903
Gender (female, %)#	58	53	0.063
Body Mass Index*	21.9 ± 2.9	19.8 ± 1.6	0.055
Smoking history (>10 packs/year, *n*)*	11	13	0.603
Atopic status (*n*)*	15	21	0.804
Asthma diagnosis (*n*)*	31	37	–
Duration of asthma (years)*	5.2 ± 4.8	4.1 ± 2.7	0.466
Presence of rhinosinusitis (%)#	91	93	0.828
Peripheral eosinophil count*	5.8 ± 5.2	20.3 ± 9.8	**0.000**
Sputum eosinophil percent (%)#	5.4 ± 8.0	40.7 ± 30.1	**0.000**
**Parameters of lung function**			
⋅FEV1% Pred*	76.3 ± 22.6	57.7 ± 18.5	**0.030**
⋅FVC % Pred*	87.3 ± 21.2	82.1 ± 15.9	0.497
⋅FEV1/FVC*	77.8 ± 18.9	55.8 ± 15.9	0.066
⋅PEF% Pred*	77.5 ± 35.7	61.0 ± 11.7	0.158
⋅MMEF75/25*	22.6 ± 22.7	27.6 ± 22.7	0.526
FeNO (ppb)*	39.3 ± 26.3	87.6 ± 53.2	**0.015**
**Organ involvement**			
⋅Arthralgia/Arthritis, n (%)#	0	0	1.000
⋅Myalgia/Myostits, n (%)#	3 (9.6)	3 (8.8)	0.959
⋅Skin, *n* (%)#	12 (38.7)	19 (53.4)	0.087
⋅Ear/nose/throat, *n* (%)#	128 (80.6)	31 (83.8)	0.828
⋅Cardiovascular Events, *n* (%)#	0 (0)	3 (8.1)	0.060
⋅Gastrointestinal, *n* (%)#	5 (16.1)	10 (27.2)	0.128
⋅Pulmonary, *n* (%)#	31 (100)	37 (100)	–
⋅Kidney, *n* (%)#	3 (9.6)	5 (13.5)	0.603
⋅Peripheral neuropathy, *n* (%)#	5 (16.1)	5 (13.5)	0.939
BVAS score*	6 ± 2.95	20.0 ± 3.7	**0.000**

Data expressed as mean ± standard deviation, median (interquartile range) or number (%). The *P*-value was calculated from the χ2 test and ANOVA, between the 2 groups. FVC, forced vital capacity; FEV1, forced expiratory volume in 1 s; PEF% Pred, percent of predicted peak expiratory flow; MMEF75/25, maximum mid expiratory flow; FeNO, fractional exhaled nitric oxide; BVAS, Birmingham Vasculitis Activity Score. *Values are expressed as means ± SEM, ^#^Values are expressed as percentage. Bold values means *P*-value < 0.05.

### Network-Based Analysis

**FIGURE 1:**
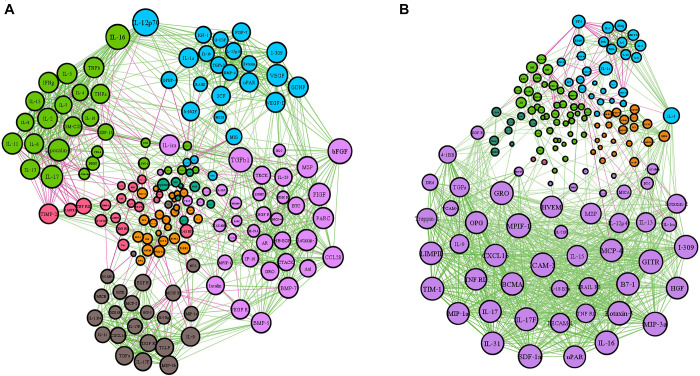
Network-based analysis of protein expression in active and inactive EGPA. The network graphs were constructed with the expression data of 160 proteins. Each protein is represented by a specific node whose color represents a specific cluster. Different colors represent the different weighting degrees of nodes and edges. The size of the node is proportional to the to the number of connections (i.e., degree). Edges between nodes represent a statistically significant association (*P* < 0.05). Edge thickness represents the strengths of association between two nodes (correlation coefficient, Spearman’s correlation). The correlation coefficient is represented by a color code, green and purple indicating positive and negative relationships, respectively. **(A)** Inactive EGPA; **(B)** Active EGPA

The patterns of serum proteins in active and inactive EGPA patients were characterized by network-based analysis (Figure 1). The active network graphs comprise 152 nodes, each representing a serum protein concentration, and a total of 1174 links representing correlations between nodes scoring a *P*-value < 0.05. The active EGPA network contains 4 clusters of highly interlinked nodes: cluster 1 gathers 51 nodes (purple) corresponding to 51 serum proteins (Supplementary Table 2); cluster 2, 21 nodes (or serum proteins; orange), cluster 3, 28 nodes (or serum proteins; blue); and cluster 4, 42 nodes (or serum proteins; green). The inactive network graphs comprise 160 nodes, each representing a serum protein concentration, and a total of 1209 links representing correlations between nodes scoring a *P*-value < 0.05. The inactive EGPA network contains 4 clusters of highly interlinked nodes: cluster 1 gathers 37 nodes (purple) corresponding to 37 serum proteins (Supplementary Table 3); cluster 2, 23 nodes (or serum proteins; brown), cluster 3, 24 nodes (or serum proteins; blue); and cluster 4, 27 nodes (or serum proteins; green). In active EGPA, cluster 1 has the largest number of nodes, suggesting that the proteins in cluster 1 are most closely related to other clusters, which may be related to disease activity. Specifically, featured cytokines associate with endothelial (PECAM-1, HVEM, VCAM-1, ICAM-3), immune cells including macrophage (CXCL16, MIP-3a, MPIF1, MIP-3b, MCP-4), eosinophil (Eotaxin-2, Eotaxin-3, CCL28), lymphocyte (TARC, GITR, IL-31, IL-17F, IL-9, IL-17, IL-16, IL-15, IL-13, IL-10 Rb, IL-18 BPa, IL21R) in cluster1 might robustly suggest that EGPA active stage involves the activation of multiple immune cells and vascular endothelium. But in inactive EGPA, the size of the nodes in the four clusters is similar, which indicates that during the inactive stage the proteins in the clusters are in balance with each other and might maintain the body’s homeostasis.

### Comparison of the Serum Levels of 160 Proteins in Inactive and Active EGPA

**FIGURE 2:**
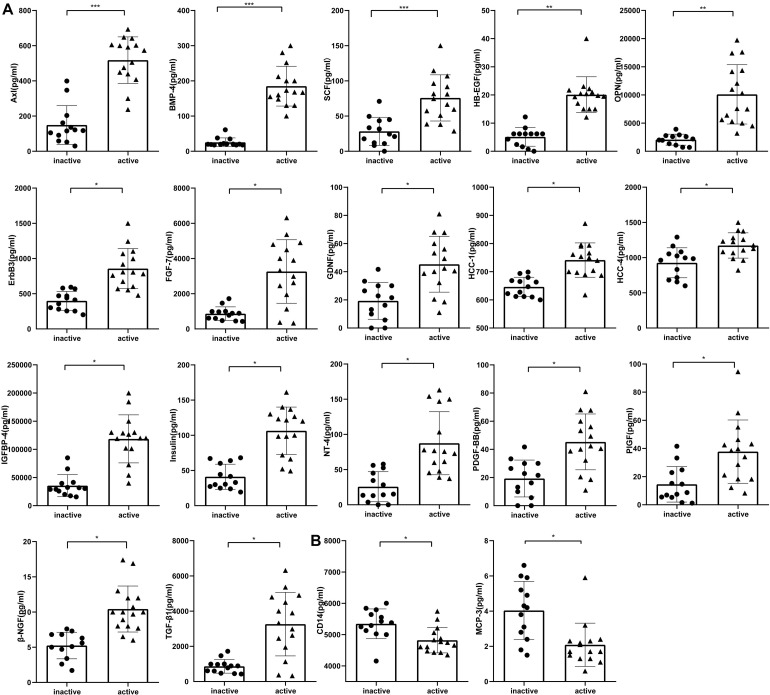
Serum levels of proteins differentially expressed in inactive and active EGPA assessed by antibody-based protein microarray. **(A)** Serum concentration of seventeen proteins were higher in active EGPA than that in inactive EGPA. **(B)** Two proteins were higher in inactive EGPA than that in active EGPA. Statistical significance was calculated by unpaired nonparametric Mann-Whitney test. * p.adj < 0.05, ** p.adj < 0.01, and *** p.adj < 0.001 indicates statistical significance between two groups (FDR correction). Data were present as Median with interquartile range. Dot symbols: inactive disease; triangle symbols: active disease.

**FIGURE 3:**
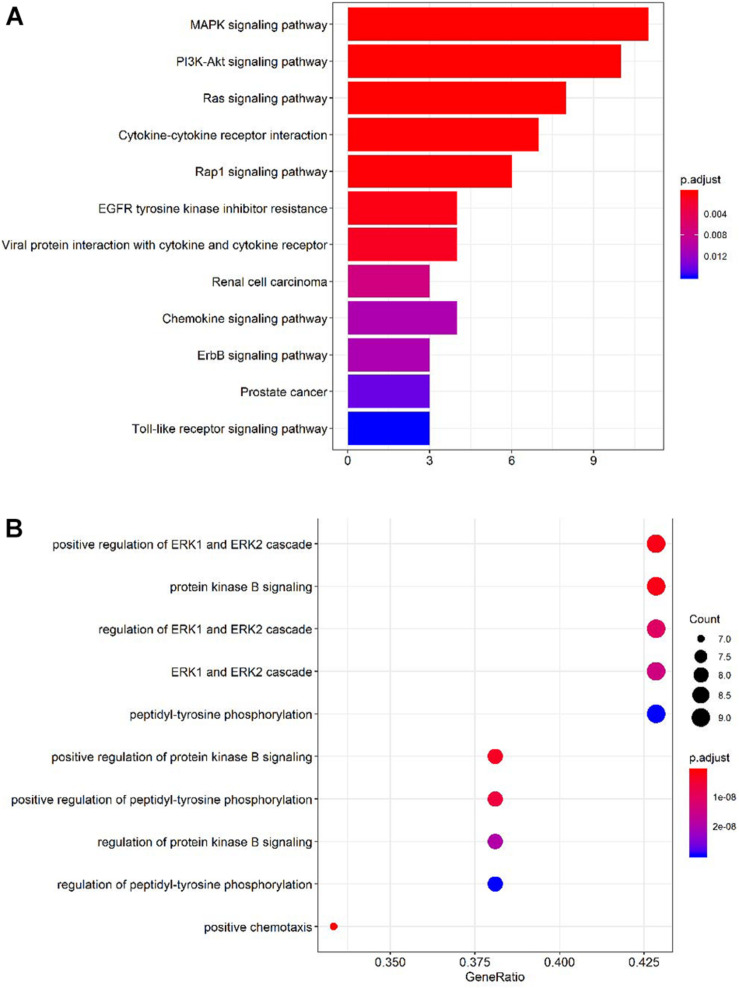
Gene Ontology (GO) and Kyoto Encyclopedia of Genes and Genomes (KEGG) pathway enrichment analyses of the proteins differentially expressed in active and inactive EGPA. **(A)** Top 10 KEGG pathways represented by the DEPs. **(B)** Top 10 GO Term biological processes represented by the DEPs.

To identify accurate and predictive biomarkers of disease activity, we first compared the serum levels of 160 serum proteins in active and inactive EGPA. Twenty-one serum proteins displayed differential levels between active and inactive disease (*P* < 0.05) (Supplementary Table 4, unpaired nonparametric Mann-Whitney test). To further strengthen our selection criteria, *P*-values were corrected using the Benjamini-Hochberg false discovery rate (FDR). Finally, 19 DEPs were identified (Figure 2). These DEPs can mainly be divided into three categories: growth factors and related receptors (BMP4, SCF, HB-EGF, OPN, FGF-7, TGF-β, IGFBP4, insulin, PDGF-BB, PIGF, ErbB3), many of which have firmly correlation with vascular remodeling; neurotrophic factor (GDNF, NT-4, β-NGF) and chemokines (HCC-1; HCC-4, CD14, MCP-3). The results probably demonstrated that multifaceted biological events engaged including neurotrophic, immune cells, and vascular activation during active disease phage. Following via using short-listed proteins, i.e., scoring FDR < 0.5, we performed KEGG pathway and GO enrichment analyses to gain insights into the potential functions involved in the clinical evolution of EGPA. Five major signaling pathways were represented by the DEPs during active EGPA, i.e., MAPK signaling pathway, PI3K-Akt signaling pathway, RAS signaling pathway, cytokine-cytokine receptor interaction and Rap1 signaling pathway. Four major biological processes were found, i.e., positive regulation of ERK1 and ERK2 cascade, protein kinase B signal, regulation of ERK1 and ERK2 cascade, ERK1 and ERK2 cascade and positive regulation of peptide-tyrosine phosphorylation (Figure 3).

### Correlations Between the Serum Level of the DEPs and Peripheral Eosinophil Cell Count in Patients With Inactive and Active EGPA

**FIGURE 4:**
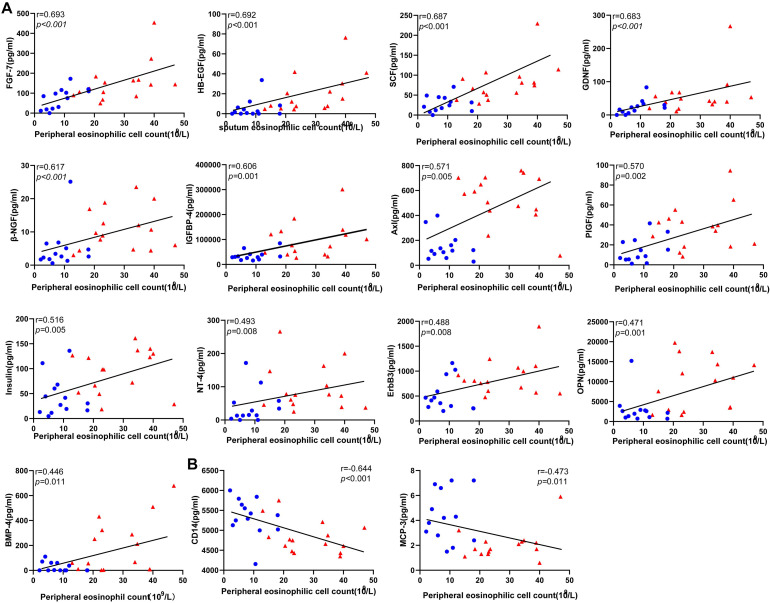
Correlations between the level of the differentially expressed protein candidates and peripheral eosinophil count in EGPA patients (Spearman correlation). Only the significant correlations are shown in dot plots. Fifteen protein markers had serum levels correlated with eosinophil cell counts. **(A)** Thirteen protein markers had a level of expression positively correlated with blood eosinophil count. **(B)** Two protein markers had level of expression negatively correlated with blood eosinophil count. Blue dots represent data from patients with inactive disease. Red triangles represent data from patients with active disease. *P*-values and Spearman correlation coefficients are displayed.

To evaluate the clinical relevance of the variation in serum level of the candidate proteins, we performed a correlation analysis between protein levels and peripheral eosinophil counts in all patients. Importantly, 15 serum proteins were significantly correlated with peripheral eosinophil count (*P* < 0.05) (Figure 4).Precisely, 13 of the proteins positively correlated with eosinophil count, while another two proteins (CD14 and MCP3) negatively correlated with eosinophil count. Results were objectively credible because CD14 and MCP3 are two chemokines with monocyte/macrophage. And The results might suggest that eosinophils participated in the process of vascular remodeling during EGPA active phage.

### Correlations Between the Serum Level of the DEPs and BVAS With Inactive and Active EGPA

**FIGURE 5:**
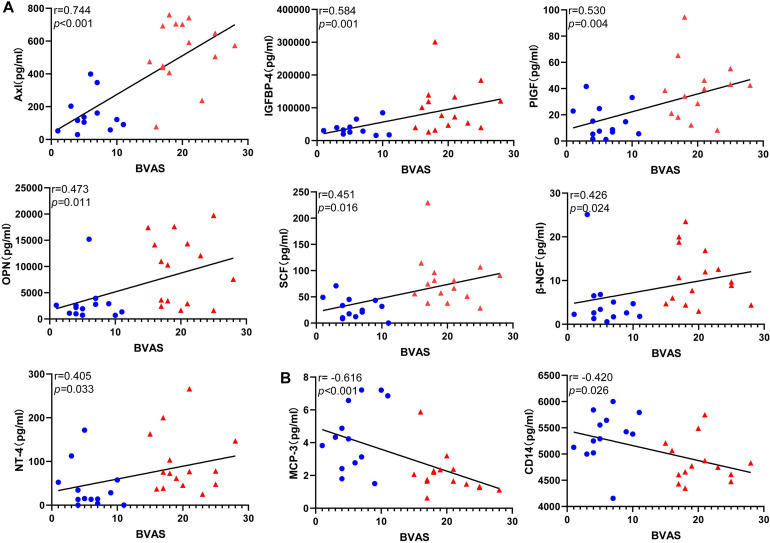
Correlations between the level of the differentially expressed protein candidates and BVAS(Spearman correlation). Only the significant correlations are shown in dot plots. Fifteen protein markers had serum levels correlated with BVAS **(A)** Thirteen protein markers had a level of expression positively correlated with BVAS. **(B)** Two protein markers had level of expression negatively correlated with BVAS. Blue dots represent data from patients with inactive disease. Red triangles represent data from patients with active disease. *P*-values and Spearman correlation coefficients are displayed.

To evaluate the relationship between DEPs and disease activity, we performed a correlation analysis between protein levels and BVAS in all patients. Nine the DEPs were significantly correlated with BVAS(*P* < 0.05) (Figure 5). Serum level of Axl, IGFBP4, PIGF, OPN, SCF, β-NGF, and NT-4 (Figure 5A)positively correlated with BVAS, while MCP-3 and CD14 (Figure 5B) negatively.

### Evaluation of the Sensitivity and Specificity of the Candidate Protein Biomarkers

**FIGURE 6:**
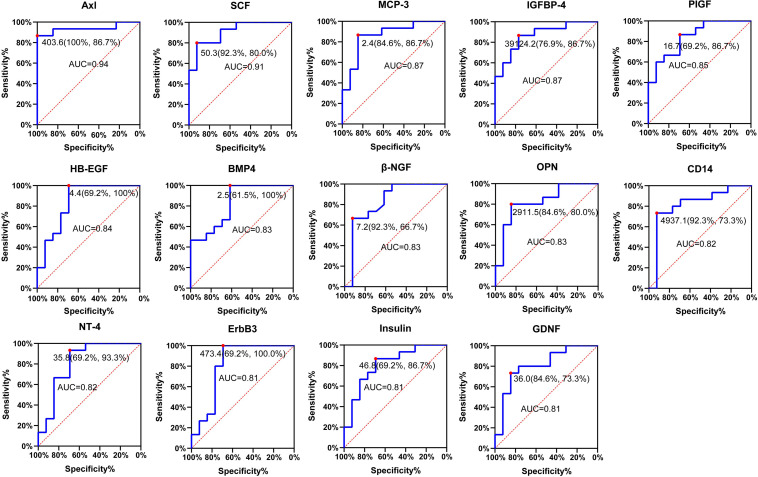
Receiver operating characteristic (ROC) analysis of candidate proteins. ROC curve showing the sensitivity and specificity for various cutoff values of the protein. Red dot indicates sensitivity at the specificity. ROC, receiver operating characteristic; AUC, area under the ROC curve.

Receiver operating characteristic (ROC) analysis is the standard approach to evaluate the sensitivity and specificity of diagnostic procedures (Song, 1997). We evaluated the sensitivity and specificity of the 15 differentially expressed serum proteins by ROC curve analysis (*P* < 0.001) (Figure 6; Supplementary Table 5). Axl has the largest AUC (0.94), specificity was 100% and sensitivity was 86.7%.

### Validation of the Differential Expression of the Biomarker Candidates by ELISA and Custom Multiplex Bio-plex Assay

**FIGURE 7:**
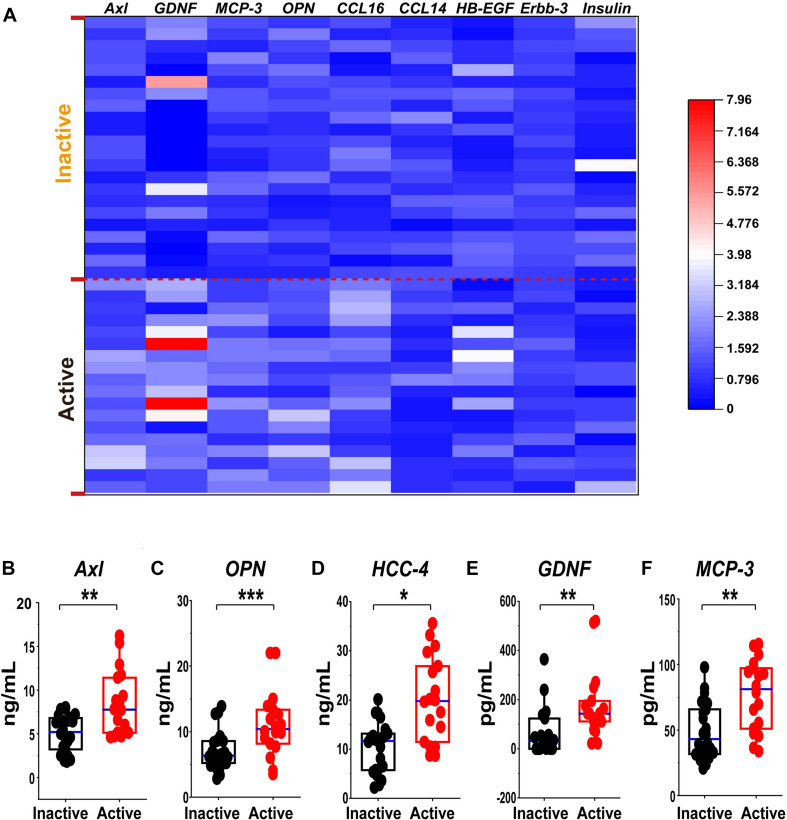
Validation of the differential expression of the serum protein marker candidates for active GEPA by ELISA and Custom Multiplex. **(A)** Heatmap comparison of the expression level of the serum protein marker candidates in inactive and active EGPA by ELISA and Bio-plex. Nine of 13 were highly expressed. **(B-F)** Scatter plots representing the median values of the expression level of the serum protein marker candidates in inactive and active EGPA assessed ELISA and Bio-plex. Five of 9 were significant difference in the group. The P values were calculated by nonparametric t-test and are noted *P < 0.05, **P < 0.01, ***P < 0.001, versus inactive group. Black dots represent data from patients with inactive disease. Red dots represent data from patients with active disease.

To further consolidate the data on the variation of candidate biomarkers levels between inactive and active EGPA, we measured serum levels of the 19 DEPs by ELISA and Custom Multiplex assay (Figure 7A). The variation of serum levels of Axl, GDNF, HCC-4, MCP-3 and OPN were consistent with the array results, confirming their differential expression in inactive and active EGPA (Figures 7B–F).

### Follow-up of Serum Level of Axl

**FIGURE 8:**
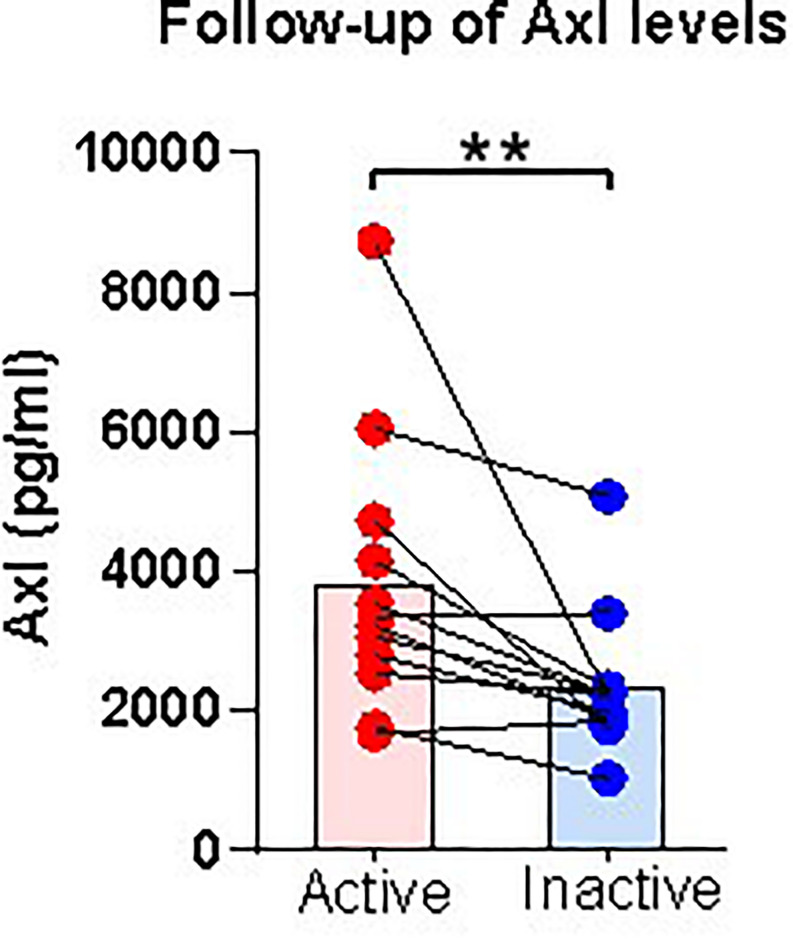
Follow-up serum level of Axl in patients after treatment. Serum levels of Axl in paired active and inactive patients. Axl serum levels were significantly diminished in inactive patients. The *P*-values were calculated by nonparametric t-test and are noted **P < 0.01, versus inactive group. Red dots represent data from patients with active disease. Blue dots represent data from patients with inactive disease.

Furthermore, to confirm the faithfully clinical diagnosis significance of Axl, we followed up the longitudinal Axl expressions for paired 12 enrolled patients in active and remission disease stage. Specifically, the serum Axl levels decreased significantly in the inactive disease compared with the baseline. Moreover, 10/12 patients showed descending tendency, suggesting that the high expression of Axl is related to the disease activity.(Figure 8).

## Discussion

In EGPA, identifying active disease is often challenging, especially when non-specific symptoms occur, such as worsening asthma or rhinosinusitis. The development of predictive biomarkers would greatly improve the ability to identify patients likely to enter an active phase of the disease. To this aim, we screened 160 soluble proteins to identify those presenting differential serum levels in inactive and active EGPA, and tested their associations with clinical characteristics. Network-based analysis defined 4 clusters of serum proteins characteristic of patients with active disease, and containing 51, 21, 28, and 42 serum proteins, respectively. Among the 160 screened proteins, 19 were significantly upregulated in the sera of patients with active disease compared with sera from patients in the inactive phase (*P* < 0.05). Spearman’s correlation analysis indicated that 16 of these DEFs were significantly correlated with peripheral eosinophil count in active and inactive EGPA patients (*P* < 0.05). Finally, we verified the differential levels of the candidate markers by ELISA and bio-plex assay. With these methods, Axl, OPN, HCC-4, GDNF and MCP-3 were further validated, and among them, Axl scored the largest AUC, indicating its superior diagnostic value above the other candidates. Thus, our study uncovered Axl as a candidate biomarker to discriminate active from inactive disease in EGPA.

Eosinophilic granulomatosis with polyangiitis is considered a Th2-mediated disease. Previous studies on the differential expression of serum ECP, CCL17/TARC, eotaxin-3, periostin and IgG4, representing Th2 features, were reported to correlate with disease activity, but could not distinguish between different phases of the disease (Guilpain et al., 2007; Dallos et al., 2010; Vaglio et al., 2012; Rhee et al., 2018). The clinical value of absolute eosinophil count, serum IgE, ESR and CRP also showed their limitations as longitudinal biomarkers of disease activity or predictors of flare in EGPA (Grayson et al., 2015). Therefore, it is urgent to uncover new serum biomarkers of active and inactive EGPA. The clinical phenotype of EGPA cannot be explained by exaggerated Th2 response alone. Pagnoux et al. (2019) explored a panel of 54 serum cytokines and chemokines involved in Th1/Th2, Th9/Th17/Th22/Treg, and inflammatory responses, but did not detect clear differences in the serum levels of these proteins, indistinguishable between active or inactive EGPA. Some of the non-differential proteins found in this previous study were also non-differential in our study. However, contrary to what Pagnoux and colleagues reported, we found the MCP-3 level was significantly different between active and inactive EGPA. The reason for this discrepancy remains unclear. In our study, all patients were treated with glucocorticoid, and 75% (51 / 68) of them were treated with glucocorticoid combined with immunosuppressors. In the cohort studied by Pagnoux et al. (2019), only 45% (18/40) of the patients were treated with glucocorticoids. These different treatments likely affected the expression of serum cytokines. In addition, ethnic differences cannot be excluded to explain the different results. Our findings also suggest that, whereas most emphasis has been given to eosinophils and some cytokines in the pathophysiology of EGPA, more attention should be paid to a possible role of monocytes/macrophages. Decision making for treatments should not rely solely on cytokine level.

Network analysis was used to compare the expression profiles of 160 soluble serum proteins in patients with active and inactive EGPA. Result showed that serum samples with different disease activity have a distinguishable expression pattern, suggesting that distinct disease activity have different target proteins.

Pathogenesis of EGPA remains largely unknown. However, like many other autoimmune diseases, genetic predisposition and environmental factors appear to contribute to the development of EGPA (Nguyen and Guillevin, 2018). Eosinophilic inflammation and ANCA-mediated vasculitis are both cardinal features of EGPA (Furuta et al., 2019). Our study found that peripheral blood eosinophils, induced sputum eosinophils and FeNO were significantly higher in patients with active EGPA than in those with inactive disease. The reasons for these differences were mainly related to the pathogenesis of the disease itself. IL-5, a main eosinophil activator, seems involved in EGPA pathogenesis. IL-5 is produced by Th2 cells and induces the differentiation and maturation of human eosinophils (Sanderson, 1988). Following activation, eosinophils release granules containing stored cytotoxic proteins and toxins such as eosinophil-derived neurotoxin, major basic protein (MBP), eosinophil peroxidase, and eosinophil cationic protein (ECP), all causing tissue damage (Guilpain et al., 2006). These pathophysiological differences explain the differential network between active and inactive EGPA. There were 19 DEPs between active and inactive EGPA, and some of these DEPs were related to eosinophil count in peripheral blood. This suggests that the differences between the two groups may be related to eosinophilic inflammation. To better understand the potential biological functions of these DEPS in active EGPA, GO and KEGG pathways analyses were performed. This analysis revealed the prominence of biological pathways involving ERK1 and ERK2. These proteins are related protein-serine/threonine kinases that participate in the Ras-Raf-MEK-ERK signal transduction cascade, regulating a broad variety of processes including cell adhesion, cell cycle progression, cell migration, cell survival, differentiation, metabolism, proliferation, and transcription (Roskoski, 2012). Robins et al. (2011) proposed ERK1/ERK2 MAPK cascade as a novel and attractive target in severe asthma, because this pathway is resistant to glucocorticoids, while it contributes the inflammation by neutrophil recruitment. This observation supports that the ERK1/ERK2 MAPK cascade may be also functionally involved in the active phase of EGPA, but this hypothesis needs further confirmation. Previous studies comparing biomarkers in serum of patients with active and inactive EGPA did not address the signaling pathways and biological functions related to the different biomarkers. In this study, GO and KEGG analyses were used for the first time to further explore the biological functions and signaling pathways of DEPs, and discover the possible mechanisms and biological roles involving the cytokines in active EGPA. It is expected that follow-up on these candidate pathways in *in vitro* and *in vivo* experiments will help better explain disease etiology and variations underlying different phases of EGPA.

The concentration of Axl and IGFBP-4 were significantly correlated with eosinophil counts in peripheral blood and sputum (Supplementary Figure 1). The reasons for this difference may be explained by the fact that the samples used for differential screening were from the peripheral blood, and not from the respiratory tract. Thus, differences may exist between the pathogenesis of the acute phase in peripheral blood and in the respiratory tract. ROC analysis showed that Axl had the highest diagnostic efficiency and significantly correlation with eosinophil counts in both peripheral blood and induced sputum, and meanwhile it has a significantly correlation with BVAS. Axl serum levels were significantly diminished in follow-up inactive patients. Therefore, Axl may be a better biomarker to distinguish active and inactive EGPA. Furthermore, Axl combined with BMP-4 and SCF could improve the diagnostic sensitivity (93.3%) (Supplementary Figure 2).

Axl is a receptor tyrosine kinase that transduces signals from the extracellular matrix to the cytoplasm by binding the growth factor GAS6. Thus, Axl regulates many physiological processes including cell survival, cell proliferation, migration and differentiation (Korshunov, 2012; Brown et al., 2016). GAS6/Axl signaling plays a role in processes such as endothelial cell survival during acidification by preventing apoptosis, optimal cytokine signaling during human natural killer cell development, hepatic regeneration, gonadotropin-releasing hormone neuron survival and migration, platelet activation, or regulation of thrombotic responses (Sasaki et al., 2006). The mechanism underlying Axl upregulation is not clear but continuous stimulation of macrophages by apoptotic debris may increase its expression and release (Lee and Chun, 2019). Axl is critical for phagocytosis of apoptotic cells and regulation of the immune system (Di Stasi et al., 2020).

Shuai Wang et al. (2021) found that, consistent with ACE2, SARS-CoV-2 can cause infection through the Axl receptor of the lung epithelium, while knocking out Axl significantly reduces the effect of SARS-CoV-2 on H1299 lung cells and human primary lung epithelial cells. Besides, Milena S. Espindola et al. (2018) found that Axl activation is obviously related to the process of pulmonary fibrosis, and pulmonary fibrosis is significantly reduced after Axl inhibition. These studies showed that Axl plays very important roles in inflammatory diseases. Therefore, further investigations on the role of the Gas6/Axl pathway in EGPA and the development of specific antagonists targeting this pathway are necessary.

The present study has limitations. First, we limited our investigation to the study of the level of expression of 160 soluble proteins in peripheral blood of patients with active and inactive subjects. Although this approach validated our strategy of identification of a new marker for active EGPA, it could be extended to whole proteomes, using broader proteomics screening. Thus, while we identified candidate cytokine markers, our study did not provide a comprehensive view on the proteomic differences distinguishing active and inactive EGPA. Second, although we uncovered correlations between some of the differentially expressed proteins and clinical features, we did not further explore the molecular mechanisms linking the protein markers to the pathology. Functional approaches will be endeavored in the future, using a broader range of clinical samples (e.g., sputum, bronchoalveolar lavage, *etc.*), to further verify the consistency of the results, and validate the use of the biomarker candidates in diagnostic and prognosis. The development of *in vitro* models and/or *in vivo* animal models of the disease will help verify the reliability of the functional results.

In conclusion, we identified a new cytokine, Axl, related to active EGPA, and demonstrated a high degree of correlation between its serum level and peripheral blood and induced sputum eosinophil counts. This is the first report of this biomarker in EGPA. Further studies involving higher numbers of clinical samples will help strengthen its clinical value for the diagnosis and prognosis of active EGPA and other vasculitis.

## Data Availability Statement

The original contributions presented in the study are included in the article/Supplementary Material, further inquiries can be directed to the corresponding author/s.

## Ethics Statement

The studies involving human participants were reviewed and approved by Ethics Review Board of The First Affiliated Hospital of Guangzhou Medical University (Medical Research Ethics Review 2018, No. 35). The patients/participants provided their written informed consent to participate in this study.

## Author Contributions

JM and CD performed the experiments and drafted the manuscript. SW, MQ, PW, CO, and BZ interpreted the patient data and collected the samples. XZ and JY co-designed the experiment strategy. NZ and QZ critically revised the manuscript. All authors read and approved the final manuscript.

## Conflict of Interest

The authors declare that the research was conducted in the absence of any commercial or financial relationships that could be construed as a potential conflict of interest. The handling editor declared a past collaboration with several of the authors MQ, NZ, and QZ.

## Footnotes

1 https://david.ncifcrf.gov/

2 https://gephi.org/

## Supplementary Material

Supplementary Figure 1 Correlations between Axl or IGFBP-4 protein levels and sputum eosinophilic cell count in EGPA patients (Spearman correlation). Only the significant correlations are shown in dot plots. Two protein markers had serum levels correlated with eosinophil cell counts. Blue dots represent data from patients with inactive disease. Red triangles represent data from patients with active disease. P values and Spearman correlation coefficients are displayed.

Supplementary Figure 2 ROC combination analysis of Axl, BMP-4 and SCF. Combination ROC curve showing the sensitivity and specificity for cutoff values of Axl with BMP-4 and SCF. Red dot indicates sensitivity at the specificity. ROC, receiver operating characteristic; AUC, area under the ROC curve.

Supplementary Table 1 Information of 160 soluble proteins.

Supplementary Table 2 The nodes or serum proteins of each cluster of network in active EGPA.

Supplementary Table 3 The nodes or serum proteins of each cluster of network in inactive EGPA.

Supplementary Table 4 Differentially expressed proteins from unpaired nonparametric Mann-Whitney test.

Supplementary Table 5 Summary of the ROC curve analysis.
