# Supplementary figures and images for: Serum Cytokine Profiling Identifies Axl as a New Biomarker Candidate for Active Eosinophilic Granulomatosis With Polyangiitis
Source: Front Mol Biosci. 2021 Apr 27;8:653461. doi: 10.3389/fmolb.2021.653461 (PMC8112820; doi:10.3389/fmolb.2021.653461)

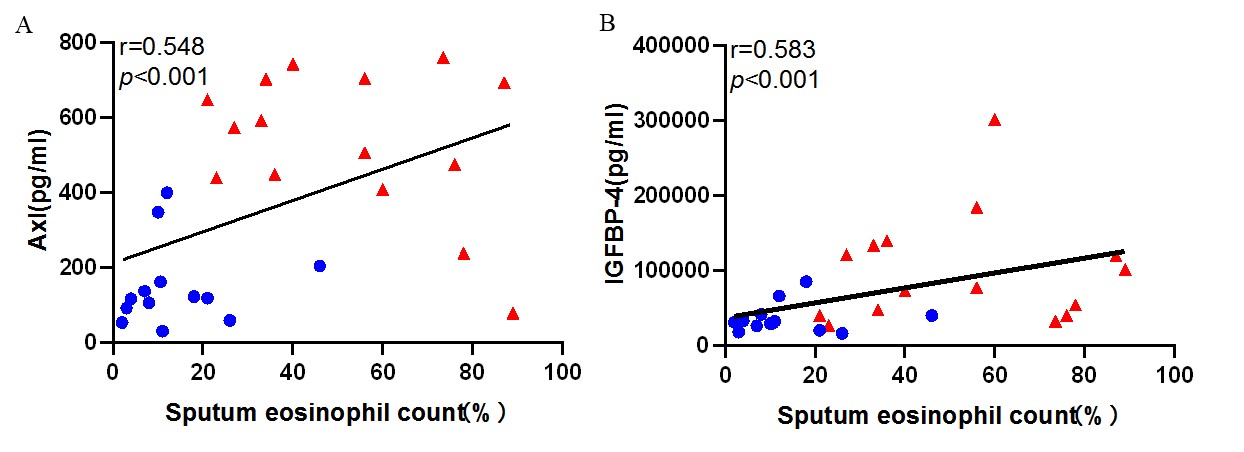

Supplement: Supplementary Figure 1 — Correlations between Axl or IGFBP-4 protein levels and sputum eosinophilic cell count in EGPA patients (Spearman correlation). Only the significant correlations are shown in dot plots. Two protein markers had serum levels correlated with eosinophil cell counts. Blue dots represent data from patients with inactive disease. Red triangles represent data from patients with active disease. P values and Spearman correlation coefficients are displayed. [file Image_1.jpg]

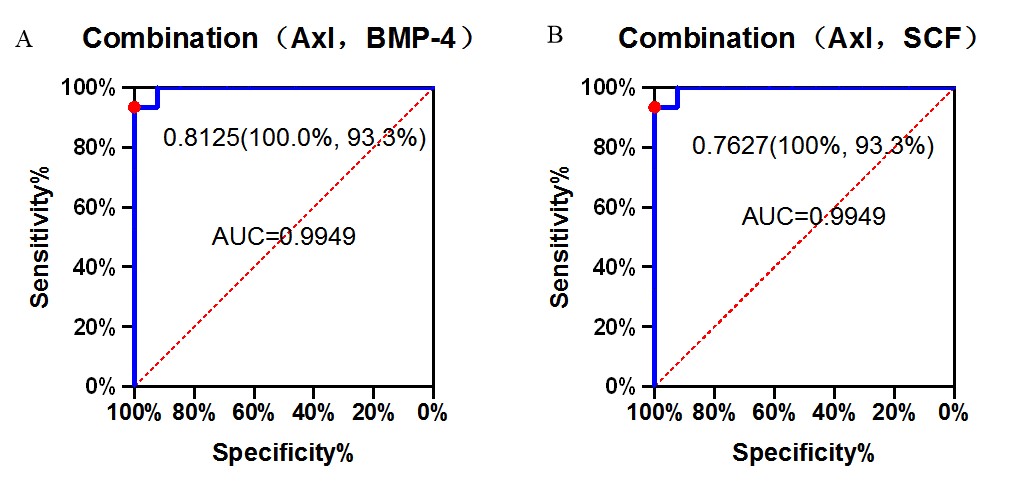

Supplement: Supplementary Figure 2 — ROC combination analysis of Axl, BMP-4 and SCF. Combination ROC curve showing the sensitivity and specificity for cutoff values of Axl with BMP-4 and SCF. Red dot indicates sensitivity at the specificity. ROC, receiver operating characteristic; AUC, area under the ROC curve. [file Image_2.jpg]
