# Supplementary material for: Serum Cytokine Profiling Identifies Axl as a New Biomarker Candidate for Active Eosinophilic Granulomatosis With Polyangiitis
Source: Front Mol Biosci. 2021 Apr 27;8:653461. doi: 10.3389/fmolb.2021.653461 (PMC8112820; doi:10.3389/fmolb.2021.653461)
Supplement: Supplementary Table 1 — Information of 160 soluble proteins. [file Table_1.doc]

**Supplementary Table 1. Information of 160 soluble proteins**

| **Symbol** | **Description** |
| --- | --- |
| 4-1BB | T-cell antigen 4-1BB |
| 6Ckine | 6Ckine |
| ALCAM | Activated leukocyte cell adhesion molecule |
| Amphiregulin | Amphiregulin |
| AXL | Tyrosine-protein kinase receptor UFO |
| BCMA | Tumor necrosis factor receptor superfamily member 17 |
| BDNF | Brain-derived neurotrophic factor |
| Betacellulin | Probetacellulin [Cleaved into: Betacellulin,BTC] |
| beta-NGF | Beta-nerve growth factor |
| bFGF | Basic fibroblast growth factor receptor 1 |
| BLC | B lymphocyte chemoattractant |
| BMP-4 | Bone morphogenetic protein 4 |
| BMP-5 | Bone morphogenetic protein 5 |
| BMP-7 | Bone morphogenetic protein 7 |
| CCL28 | C-C motif chemokine 28 |
| CD14 | Monocyte differentiation antigen CD14 |
| CD30 | Lymphocyte activation antigen CD30 |
| CD40 Ligand | CD40 ligand |
| CD80 | T-lymphocyte activation antigen CD80 |
| CEACAM-1 | Carcinoembryonic antigen-related cell adhesion molecule 1 |
| Contactin-2 | Contactin-2 |
| CTACK | Cutaneous T-cell-attracting chemokine |
| CXCL16 | C-X-C motif chemokine 16 |
| DR6 | Death receptor 6 |
| Dtk | Tyrosine-protein kinase receptor TYRO3 |
| EGF | Epidermal growth factor |
| EGFR | Epidermal growth factor receptor |
| EG-VEGF | Endocrine-gland-derived vascular endothelial growth factor |
| ENA-78 | Epithelial-derived neutrophil-activating protein 78 |
| Endoglin | Endoglin |
| Eotaxin-1 | Eotaxin |
| Eotaxin-2 | Eotaxin-2 |
| Eotaxin-3 | Eotaxin-3 |
| ErbB3 | Receptor tyrosine-protein kinase erbB-3 |
| E-Selectin | E-Selectin |
| Fas | Apoptosis-mediating surface antigen FAS |
| FGF-4 | Fibroblast growth factor 4 |
| FGF-7 | Fibroblast growth factor 7 |
| Flt-3 Ligand | Fms-related tyrosine kinase 3 ligand |
| GCP-2 | Granulocyte chemotactic protein 2 |
| GCSF | Granulocyte colony-stimulating factor |
| GDF-15 | Growth/differentiation factor 15 |
| GDNF | Glial cell line-derived neurotrophic factor |
| GITR | Glucocorticoid-induced TNFR-related protein |
| GM-CSF | Granulocyte-macrophage colony-stimulating factor |
| GRO alpha/beta/gamma | Growth-regulated protein alpha/beta/gamma |
| Growth Hormone | Growth Hormone |
| HB-EGF | Heparin-binding EGF-like growth factor |
| HCC-1 | Hepatocellular carcinoma protein 1 |
| HCC-4 | Hepatocellular carcinoma protein 4 |
| HGF | Hepatocyte growth factor |
| HVEM | Herpes virus entry mediator |
| I-309 | T lymphocyte-secreted protein I-309 |
| ICAM-1 | Intercellular adhesion molecule 1 |
| ICAM-3 | Intercellular adhesion molecule 4 |
| IFN-gamma | Interferon gamma |
| IGF-1 | Insulin-like growth factor I |
| IGFBP-1 | Insulin-like growth factor-binding protein 1 |
| IGFBP-2 | Insulin-like growth factor-binding protein 2 |
| IGFBP-3 | Insulin-like growth factor-binding protein3 |
| IGFBP-4 | Insulin-like growth factor-binding protein4 |
| IGFBP-6 | Insulin-like growth factor-binding protein6 |
| IL-1 alpha | Interleukin-1 alpha |
| IL-1 beta | Interleukin-1 beta |
| IL-1 R1 | Interleukin-1 receptor type 1 |
| IL-1 Ra | Interleukin-1 receptor antagonist protein |
| IL-10 | Interleukin-10 |
| IL-10 R beta | Interleukin-10 receptor subunit beta |
| IL-11 | Interleukin-11 |
| IL-12 p40 | Interleukin-12 subunit p40 |
| IL-12 p70 | Interleukin-12 subunit p70 |
| IL-13 | Interleukin-13 |
| IL-15 | Interleukin-15 |
| IL-16 | Interleukin-16 |
| IL-17 RA | Interleukin-17 receptor A |
| IL-17A | Interleukin-17A |
| IL-17F | Interleukin-17 F |
| IL-18 BP alpha | Interleukin-18 binding protein alpha |
| IL-2 | Interleukin-2 |
| IL-2 R gamma | Interleukin-2 receptor subunit gamma |
| IL-21 R | Interleukin-21 receptor |
| IL-28A | Interleukin-28A |
| IL-29 | Interleukin-29 |
| IL-31 | Interleukin-31 |
| IL-4 | Interleukin-4 |
| IL-5 | Interleukin-5 |
| IL-6 | Interleukin-6 |
| IL-6 R | Interleukin-6 recepto |
| IL-7 | Interleukin-7 |
| IL-8 | Interleukin-8 |
| IL-9 | Interleukin-9 |
| Insulin | Insulin |
| IP-10 | 10 kDa interferon gamma-induced protein |
| I-TAC | Interferon-inducible T-cell alpha chemoattractant |
| LIF | Leukemia inhibitory factor |
| LIGHT | Tumor necrosis factor ligand superfamily member 14 |
| LIMPII | Lysosome membrane protein II |
| Lipocalin-2 | Lipocalin-2 |
| L-Selectin | L-Selectin |
| Lymphotactin | Lymphotactin |
| LYVE-1 | Lymphatic vessel endothelial hyaluronic acid receptor 1 |
| MCP-1 | Monocyte chemotactic protein 1 |
| MCP-2 | Monocyte chemotactic protein 2 |
| MCP-3 | Monocyte chemotactic protein 3 |
| MCP-4 | Monocyte chemotactic protein 4 |
| M-CSF | Macrophage colony-stimulating factor |
| M-CSF R | Macrophage colony-stimulating factor receptor |
| MDC | Macrophage-derived chemokine |
| MICA | MHC class I polypeptide-related sequence A |
| MICB | MHC class I polypeptide-related sequence B |
| MIF | Macrophage migration inhibitory factor |
| MIG, | Monokine induced by interferon-gamma |
| MIP-1 alpha | Macrophage inflammatory protein 1 alpha |
| MIP-1 beta | Macrophage inflammatory protein 1 beta |
| MIP-1 delta | Macrophage inflammatory protein 1 delta |
| MIP-3 alpha | Macrophage inflammatory protein 3 alpha |
| MIP-3 beta | Macrophage inflammatory protein 3 beta |
| MPIF-1 | Myeloid progenitor inhibitory factor 1 |
| MSP alpha/beta | Macrophage-stimulating protein alpha/beta |
| NAP-2 | Neutrophil-activating peptide 2 |
| NGFR | Nerve growth factor receptor |
| NRG1-beta 1 | Neuregulin-1 beta 1 |
| NT-3 | Neurotrophin-3 |
| NT-4 | Neurotrophin-4 |
| Osteopontin (OPN) | Osteopontin |
| Osteoprotegerin (OPG) | Osteoprotegerin |
| PARC | Pulmonary and activation-regulated chemokine |
| PDGF R beta | Beta platelet-derived growth factor receptor |
| PDGF-AA | Platelet-derived growth factor AA |
| PDGF-BB | Platelet-derived growth factor BB |
| PECAM-1 | Platelet endothelial cell adhesion molecule1 |
| Platelet Factor 4 | Platelet Factor 4 |
| PLGF | Placenta growth factor |
| RAGE | Renal tumor antigen |
| RANTES | T-cell-specific protein RANTES |
| SCF | Stem cell factor |
| SCF R | Stem cell factor receptor |
| SDF-1 alpha | Stromal cell-derived factor 1alpha |
| TARC | Thymus and activation-regulated chemokine |
| TECK | Thymus-expressed chemokine |
| TGF alpha | Transforming growth factor alpha |
| TGF beta 1 | Transforming growth factor beta 1 |
| TGF beta 3 | Transforming growth factor beta 3 |
| TIM-1 | T-cell immunoglobulin mucin receptor 1 |
| TIMP-1 | Tissue inhibitor of metalloproteinases 1 |
| TIMP-2 | Tissue inhibitor of metalloproteinases 2 |
| TNF alpha | Tumor necrosis factor alpha |
| TNF beta | Tumor necrosis factor beta |
| TNF RI | Tumor necrosis factor receptor type I |
| TNF RII | Tumor necrosis factor receptor typeII |
| TRAIL R3 | TNF-related apoptosis-inducing ligand receptor 3 |
| Trappin-2 | Trappin-2 |
| TSLP | Thymic stromal lymphopoietin |
| uPAR | Urokinase plasminogen activator surface receptor |
| VCAM-1 | Vascular cell adhesion protein 1 |
| VEGF-A | Vascular endothelial growth factor A |
| VEGF-D | Vascular endothelial growth factor D |
| VEGFR2 | Vascular endothelial growth factor receptor 2 |
