# Supplementary material for: Serum Cytokine Profiling Identifies Axl as a New Biomarker Candidate for Active Eosinophilic Granulomatosis With Polyangiitis
Source: Front Mol Biosci. 2021 Apr 27;8:653461. doi: 10.3389/fmolb.2021.653461 (PMC8112820; doi:10.3389/fmolb.2021.653461)
Supplement: Supplementary Table 2 — The nodes or serum proteins of each cluster of network in active EGPA. [file Table_2.doc]

| **Supplementary Table 2.The nodes or serum proteins of each cluster of network in active EGPA** | |
| --- | --- |
|  | Proteins |
| Cluster1 | IL-31, IL-17F, TIM-1, LIMPII, TGFa, IL-9, MIP-1a, IL-17, BCMA, IL-16, Eotaxin-3, CXCL16, 4-1BB, ICAM-3, SDF-1a, OPG, MIP-3a, I-309, Trappin-2, MPIF1,  GITR, MIP-3b, B7-1, uPAR, IL-15, DR6, MCP-4, Eotaxin-2, HGF, IL-13, GRO, MSP, TARC, TNFRI, IL-12p40, PECAM-1, MICA, TNFRII, HVEM, TRAILR3,  IL-1ra, VCAM-1, CEACAM-1, BTC, IL-21R, IL-10 Rb, IL-18 BPa, EGFR, CCL28, Insulin, Dtk4 |
| Cluster2 | GDNF, FGF-7, MIG, G-CSF, ErbB3, MCSF, HB-EGF, TGFb3, IL-1b, Lymphotactin, SCF, MIP-1b, IGFBP-4, BMP-7, NGFR, MCP-1, PIGF, EG-VEGF, NT-4, GCP-2, NAP-2 |
| Cluster3 | IL-4, IL-12p70, IL-6, IFNg, TNFb, TNFa, IL-8, IL-2, IL-7, GM-CSF, IL-10, LIF, IL-28A, IL-5, MDC, PDGF-BB, IL-1a, PF4, OPN, CTACK, BDNF, PDGF-AA, BLC, ICAM-1, HCC-1, Flt-3L, MCP-2, VEGF R3 |
| Cluster4 | VEGFR2, MIF, I-TAC, GH, IGFBP-2, IGFBP-1, MCP-3, TECK, ENA-78, EGF, AR, VEGF, VEGF-D, MCSFR, IGF-1, ALCAM, Axl, FGF-4, BMP-4, SCFR, IGFBP-6, Fas, GDF-15, LYVE-1, Lipocalin-2, PARC, LIGHT, Endoglin, XEDAR,6Ckine, TGFb1, E-Selectin, IGFBP-3, NRG1-b1, Eotaxin, CD14, RANTES, bFGF, TIMP-2, NT-3, L-Selectin, IL-6R |
