# Supplementary material for: Serum Cytokine Profiling Identifies Axl as a New Biomarker Candidate for Active Eosinophilic Granulomatosis With Polyangiitis
Source: Front Mol Biosci. 2021 Apr 27;8:653461. doi: 10.3389/fmolb.2021.653461 (PMC8112820; doi:10.3389/fmolb.2021.653461)
Supplement: Supplementary Table 3 — The nodes or serum proteins of each cluster of network in inactive EGPA. [file Table_3.doc]

| **Supplementary Table 3. The nodes or serum proteins of each cluster of network in inactive EGPA** | |
| --- | --- |
|  | Proteins |
| Cluster1 | IVEGFR3, BMP-5, Insulin, BMP-7, BLC, MSP, bFGF, PIGF, MCP-4, AR, PARC, VEGF R2, IL-21R, Axl, TARC, IL-28A, Eotaxin-3, CCL28, CTACK, HB-EGF, MPIF-1, GRO, IP-10, CEACAM-1, IL-1 RI, IL-29, BTC, TGFb1, IL-1ra, Eotaxin-2, I-TAC, LIGHT, Endoglin, HCC-1, TECK, NGF R, IL-6R |
| Cluster2 | GCP-2, IL-2Rg, IL-9, MCSFR, PDGFRb, IL-1R4, TSLP, MIP-3b, IL-17R, TGFa, IL-17F,  XEDAR, IL-31, CXCL16, MIP-3a, EGFR, MCP-2, GITR, MICB, PECAM-1, NT-3, GH,  TRAIL R3 |
| Cluster3 | IGF-1, TGFb3, IL-1b, IL-12p40, FGF-7,G-CSF, BMP-4, Eotaxin,uPAR,I-309, GDNF, IL-1a,VEGF-D,IL-12p70, IGFBP-4, MCSF, b-NGF, RAGE, VEGF, OPG, MIG, SCF, FGF-4, VCAM-1 |
| Cluster4 | LYVE-1, IL-16, MDC, ICAM-1, IGFBP-3, GDF-15, MCP-1, TNFa, TNFb, IL-15, IL-7, IL-4, IL-8, GM-CSF, IL-2, IL-6, IL-5, IFNg, IL-13, IL-10, IL-11, IL-17, Lipocalin-2, CD30, BDNF, CD14, L-Selectin |
