# Supplementary material for: Serum Cytokine Profiling Identifies Axl as a New Biomarker Candidate for Active Eosinophilic Granulomatosis With Polyangiitis
Source: Front Mol Biosci. 2021 Apr 27;8:653461. doi: 10.3389/fmolb.2021.653461 (PMC8112820; doi:10.3389/fmolb.2021.653461)
Supplement: Supplementary Table 4 — Differentially expressed proteins from unpaired nonparametric Mann-Whitney test. [file Table_4.doc]

| **Supplementary Table 4. Differentially expressed proteins from unpaired nonparametric Mann-Whitney test** | |
| --- | --- |
| Protein | *P* value |
| Axl | 0 |
| SCF | 0 |
| MCP-3 | 0.017 |
| IGFBP-4 | 0.017 |
| RANTEES | 0.017 |
| PIGF | 0.024 |
| HB-EGF | 0.024 |
| OPN | 0.026 |
| BMP-4 | 0.026 |
| β-NGF | 0.026 |
| HCC-1 | 0.027 |
| HCC-4 | 0.027 |
| Insulin | 0.027 |
| NT-4 | 0.027 |
| CD14 | 0.027 |
| Erb3 | 0.027 |
| GDNF | 0.029 |
| PDGF-BB | 0.029 |
| TGFβ1 | 0.045 |
| FGF-7 | 0.049 |
| TGF-α | 0.049 |
