# Supplementary material for: Serum Cytokine Profiling Identifies Axl as a New Biomarker Candidate for Active Eosinophilic Granulomatosis With Polyangiitis
Source: Front Mol Biosci. 2021 Apr 27;8:653461. doi: 10.3389/fmolb.2021.653461 (PMC8112820; doi:10.3389/fmolb.2021.653461)
Supplement: Supplementary Table 5 — Summary of the ROC curve analysis. [file Table_5.doc]

| **Supplementary Table 5. Summary of the ROC curve analysis** | | | | | |
| --- | --- | --- | --- | --- | --- |
| Protein | Cutoff (ng/mL) | Specificity (%) | Sensitivity (%) | AUC | *P* value |
| Axl | 403.6 | 100.0 | 86.7 | 0.94 | <0.001 |
| SCF | 50.3 | 92.3 | 80.0 | 0.91 | <0.001 |
| MCP-3 | 2.4 | 84.6 | 86.7 | 0.87 | <0.001 |
| IGFBP-4 | 39714.2 | 76.9 | 86.7 | 0.87 | <0.001 |
| PIGF | 16.7 | 69.2 | 86.7 | 0.85 | <0.001 |
| HB-EGF | 4.4 | 69.2 | 100.0 | 0.84 | <0.001 |
| BMP-4 | 2.5 | 61.5 | 100.0 | 0.83 | <0.001 |
| β-NGF | 7.2 | 92.3 | 66.7 | 0.83 | <0.001 |
| OPN | 2911.5 | 84.6 | 80.0 | 0.83 | <0.001 |
| CD14 | 4937.1 | 92.3 | 73.3 | 0.82 | <0.001 |
| HCC-1 | 683.2 | 84.6 | 80.0 | 0.82 | <0.001 |
| NT-4 | 35.8 | 69.2 | 93.3 | 0.82 | <0.001 |
| ErbB3 | 473.4 | 69.2 | 100.0 | 0.81 | <0.001 |
| HCC-4 | 1082.8 | 84.6 | 73.3 | 0.81 | <0.001 |
| Insulin | 46.8 | 69.2 | 86.7 | 0.81 | <0.001 |
| GDNF | 36.0 | 84.6 | 73.3 | 0.81 | <0.001 |
| PDGF-BB | 712.7 | 61.5 | 93.3 | 0.81 | <0.001 |
| TGFb1 | 1835.9 | 84.6 | 73.3 | 0.79 | <0.001 |
| FGF-7 | 121.6 | 92.3 | 60.0 | 0.78 | <0.001 |
